# The combination of endurance exercise and SGTC (Salvia–Ginseng–Trigonella–Cinnamon) ameliorate mitochondrial markers’ overexpression with sufficient ATP production in the skeletal muscle of mice fed AGEs-rich high-fat diet

**DOI:** 10.1186/s12986-022-00652-w

**Published:** 2022-03-05

**Authors:** Maryam Haghparast Azad, Iman Niktab, Shaghayegh Dastjerdi, Navid Abedpoor, Golbarg Rahimi, Zahra Safaeinejad, Maryam Peymani, Farzad Seyed Forootan, Majid Asadi-Shekaari, Mohammad Hossein Nasr Esfahani, Kamran Ghaedi

**Affiliations:** 1 ACECR Institute of Higher Education, Isfahan, Iran; 2 Department of Animal Biotechnology, Cell Science Research Center, Royan Institute for Biotechnology, ACECR, Isfahan, Iran; 3 Department of Cell and Molecular Biology and Microbiology, Faculty of Biological Science and Technology, University of Isfahan, Hezar Jerib Ave., Azadi Sq., P.O. Code 81746-73441, Isfahan, Iran; 4 Department of Biology, Faculty of Basic Sciences, Shahrekord Branch, Islamic Azad University, Shahrekord, Iran; 5 Legal Medicine Research Center, Legal Medicine Organization, Tehran, Iran; 6 Neuroscience Research Center, Neuropharmacology Institute, Kerman University of Medical Sciences, Kerman, Iran

**Keywords:** ATP, Advanced glycation-end products, Endurance exercise, High fat diet, Mitochondria, Type 2 diabetes

## Abstract

**Background:**

Skeletal muscle mitochondria is one of the most important affected sites of T2DM and its molecular mechanism is yet to be elucidated. Some recent theories believed that mitochondrial markers are upregulated in response to high fat induced T2DM; however, the reasons and the affected factors are still uncertain. In this regard, we aimed to investigate the effect of high fat induced T2DM on mitochondrial markers of skeletal muscle, and an herbal component along with endurance exercise, as probable treatments, in AGE-rich high-fat diet (AGEs-HFD) induced T2DM mice.

**Methods:**

T2DM was induced by 16 weeks of AGEs-HFD consumption in male C57BL/6 mice, followed by 8 weeks of drugs ingestion and endurance exercise treatments (n = 6 in each group and total number of 42 mice). The herbal component was an aquatic extract of Salvia officinalis, Trigonella foenum-graecum, Panax ginseng, and Cinnamomum zeylanicum, termed “SGTC”. We then examined the relative expression of several mitochondrial markers, including Ppargc1α, Tfam, and electron transport chain genes and ATP levels, in skeletal muscle samples.

**Results:**

T2DM was successfully induced according to morphological, biochemical, and molecular observations. All mitochondrial markers, including Ppargc1a, Tfam, Cpt2, and electron transport chain genes, were upregulated in T2DM group compared to controls with no significant changes in the ATP levels. Most mitochondrial markers were downregulated by drug treatment compared to T2DM, but the ATP level was not significantly altered. All mitochondrial markers were upregulated in exercised group compared to T2DM with mild increase in the ATP level. The Ex + SGTC group had moderate level of mitochondrial markers compared to T2DM, but the highest ATP production.

**Conclusion:**

The highly significant overexpression of mitochondrial markers may be in response to free fatty acid overload. However, the lack of significant change in the ATP level may be a result of ROS generation due to electron leakage in the AGEsRAGE axis and electron transport chain. Almost all treatments ameliorate mitochondrial markers’ overexpression. The SGTC appears to regulate this with its antioxidant properties. Instead, exercise upregulated mitochondrial markers efficiently; however, the most efficient results, i.e. the most ATP production among the treatments, were observed in the Ex + SGTC group.

**Graphical Abstract:**

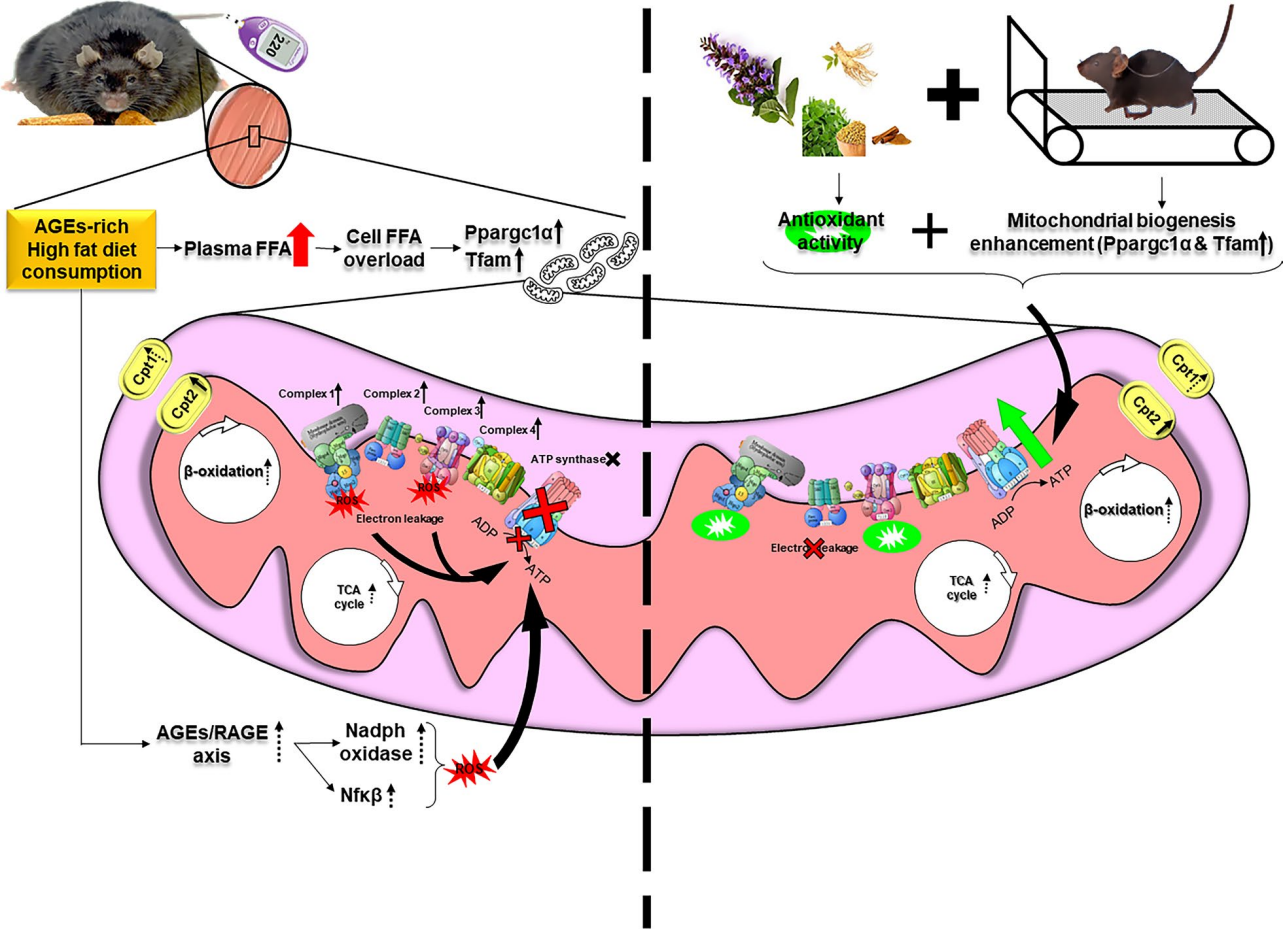

**Supplementary Information:**

The online version contains supplementary material available at 10.1186/s12986-022-00652-w.

## Background

Diabetes is becoming a pandemic concern against the human health. It is characterized by hyperglycemia, due to the development of insulin resistance (IR) in the peripheral tissues [1]. Obesity and sedentary lifestyle are supposed to be important risk factors for IR and type II diabetes mellitus (T2DM) [2]. Due to the current lifestyle, including more high fat enriched diet and processed food consumption and lower physical activity, obesity is on the rise [3]. Globalization and industrialization of food processing methods have increased the amount of advanced glycation-end products (AGEs) compounds especially in the Western diet [4]. AGEs are heterogeneous compounds that are formed mainly via Maillard reaction between sugar and protein or lipid in non-enzymatic reactions [5]. Interaction between AGEs and their receptor (RAGE) triggers inflammation and stress oxidative pathway, leading to mitochondrial dysfunction [6].

Mitochondria, as a crucial organelle for supplying adequate levels of ATP for skeletal muscle high oxidative demands, is one of the major affected sites of T2DM [7]. In the two past decades, it has been hypothesized that IR is mediated by deficiency of mitochondrial function in skeletal muscle [8]. Decreasing the capacity of fat oxidation and subsequently accumulation of intramyocellular lipids such as diacylglycerol (DAG) and ceramide (CER) finally leads to disrupting insulin signaling and IR development [8]. In contrast, recent studies have demonstrated that high fat diet (HFD) may intensify mitochondrial biogenesis -via Ppargc1α and Tfam as the main regulators of mitochondrial biogenesis –and oxidative metabolism markers by increasing fatty acid oxidative capacity to handle this situation [9]. In fact, free fatty acid overload triggers the activation of PPARγ and Ppargc1α, ultimately leading to mitochondrial gene transcription and mitochondrial biogenesis [10], which may not followed by ATP production.

Controlled Lifestyle interventions including eating habits improvement and regular exercise sessions are the first recommendation for the lifestyle-dependent disorders e.g. T2DM [11]. Besides, herbal medicines have been used for various purposes, including diabetes treatment, for a long time. Many herbal components have been shown to have hypoglycemic properties and they can play a significant role in reducing the effects of diabetes. *Salvia*, Ginseng, *Trigonella*, and Cinnamon (SGTC) are among these plants [12, 13]. These plants are also considered because of their antioxidant properties. Furthermore, regular exercise is highly recommended for diabetic patients. The beneficial effect of exercise, especially endurance exercise, in improving T2DM has been demonstrated in many studies [12–15]. It has been proven that endurance exercise increases the size and contents of mitochondria and enhances the rate of ATP production in muscle cells [14]. Additionally, it has been reported that endurance exercise regulates mitochondrial biogenesis and function through some known mechanisms [15].

The aim of our study was to examine mitochondrial response to diabetic condition, at early stage. Following that, we examined the effects of herbal component, SGTC, and endurance exercise on mitochondrial markers. The simultaneous effects of both treatments were also tested on mitochondrial function in obese C57BL/6 mice with T2DM.

## Methods

### Herbal component

The aqueous extract of the four herbs was provided as previously described [16]. The final extract was consisted of 50% (w/v) of *Salvia officinalis*, 21% (w/v) of *Panax ginseng*, 21% (w/v) of *Trigonella foenum-greacum*, and 8% (w/v) of *Cinnamomum zeylanicum*.

### Fourier-transform infrared spectroscopy (FTIR) assay of SGTC

The FTIR assay, was carried out on the SGTC to measure the antioxidant capacity of the component [17], by Nicolet™ 380 (thermos Science, USA). The results were then processed using hyperSpec package in the R programming language version 3.6.0.

### Animal model and diabetes induction

All animal research protocols in this study were approved by Royan reviewer board members and Iran National Committee for Ethics in Biomedical Research, Royan Institute-ACECR (Ethics Committee) (IR.ACECR.ROYAN.REC.1397.170, issued date: 2018-10-10). All experiment were performed in accordance to Royan institute animal research guidelines entitled “Laboratory animals’ care guideline in scientific researches” and ARRIVE guidelines 2.0 and recommendations.

**Fig. 1:**
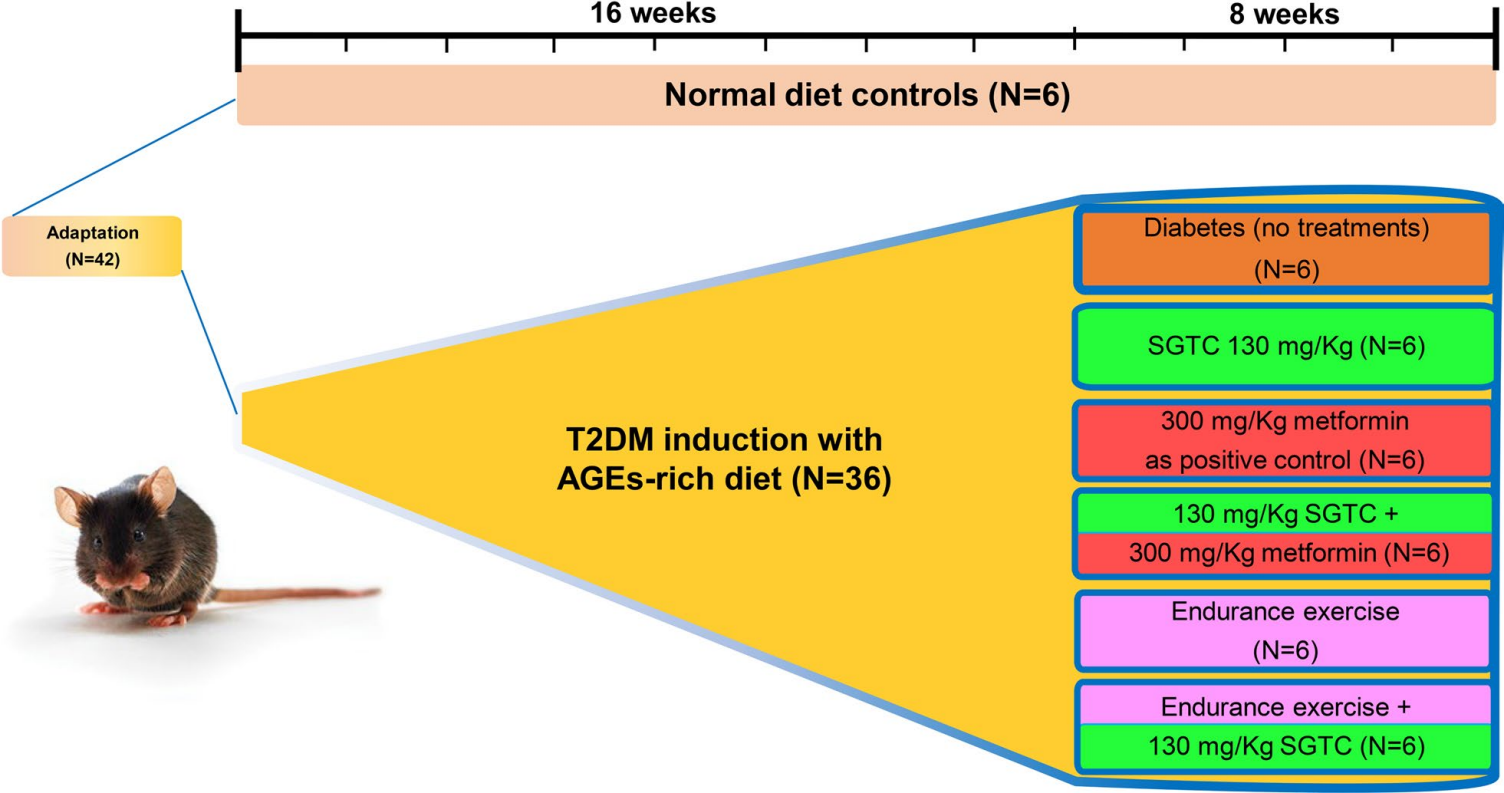
Illustrated protocol of T2DM induction and treatments in C57BL/6 mouse model. As shown in the graphical protocol the animals were randomly divided into two groups of healthy controls with normal diet consumption and type 2 diabetes induction by AGE-rich high fat diet. The animal experiment phase of this study started with 2 weeks of adaptation to the AGE-rich high fat diet and experiment condition. It was followed by 16 weeks of T2DM induction by the AGE-rich high fat diet. After confirmation of T2DM induction, the animals were randomly divided into 6 groups and 8 weeks of different treatments were applied

This study was started with 4 weeks old male C57BL/6 inbreed mice weighting 14 ± 2 g provided by Isfahan Royan Institute for biotechnology (n = 42, 6 mice/cage). Initially, mice were randomly divided into 2 main groups: Control (n = 6) and AGEs (n = 36). The control group was fed with chow diet for the rest of the experiment. The AGEs groups were fed with a combination of chow and AGE-rich HFD, which started with chow diet and gradually shifted to AGEs rich HFD within 2 weeks, for adaptation to AGE-rich HFD, then it was followed by 16 weeks of AGE-rich HFD for diabetic induction (Fig. 1). Subsequently, T2DM mice were randomly divided into 6 groups (n = 6) and 8 weeks of treatments was performed with continuing the AGEs-rich HFD feeding (Fig. 1). The treatments include:

- AGEs rich high fat diet fed mice with no additional treatments.
- AGEs rich high fat diet fed mice with oral administration of 130 mg/Kg of SGTC (Goldaru, Iran).
- AGEs rich high fat diet fed mice with oral administration of 300 mg/Kg of Metformin (Raha pharmaceutical co., Iran) as the positive control group.
- AGEs rich high fat diet fed mice with oral administration of 130 mg/Kg of SGTC along with 300 mg/Kg of Metformin.
- AGEs rich high fat diet fed mice with endurance exercise.
- AGEs rich high fat diet fed mice with oral administration of 130 mg/Kg of SGTC along with endurance exercise.

The animals were kept at 22–24 °C temperature-controlled room with 65 ± 5% humidity, and were exposed to 12/12 h light/dark cycle with free access to water and food.

Eventually, when the animals were 30 weeks old, the animals were fasted for 6 h, anesthetized, and sacrificed. Approximately, 80–100 mg/kg of Ketamine along with 5–10 mg/kg of Xylazine was injected intra-peritoneal (I.P.) for anesthetization. Fresh blood was taken from the heart of the animal. Then blood samples were centrifuged at 3000*g* for 15 min at 4 °C for serum isolation. Other tissues were removed and washed in PBS^−^ for snap freezing in − 80 °C for further investigations.

### Herbal and chemical drugs dose and administration details

The herbal and chemical compounds were administered by intra-gastric gavage once per day before refeeding. The SGTC component was administered at a dose of 130 mg/kg (~ 14.5 mg/kg/day HED) and metformin was administered at a dose of 300 mg/kg (~ 33.3 mg/kg/day HED). Endurance exercise was performed once per day before refeeding. In the case that two treatments were combined, the exercise was performed prior to drug consumption. The exercise protocol is shown in Additional file 1: Table S1 as described [18, 19].

### Fasting blood sugar (FBS) level, glucose tolerance test and serum insulin level measurement

In order to measure FBS level, mice were kept in fasting state for 6 h and the FBS level was measured by glucometer (Alpha TRAK2, USA) from a single drop of blood taken from the tail vein. The same procedure was performed after intraperitoneal injection of 1 mg/kg of D-(+)-Glucose (G7021 Sigma-Aldrich, Germany) for glucose tolerance test (GTT) and the level of blood glucose was measured before and after injection at 30, 60, 90, and 120 min of intervals by glucometer (Alpha TRAK, USA). The serum insulin level was measured by ultra-sensitive mouse insulin ELISA kit (90080 crystal-chem, USA) according to manufacturer instruction.

### Total RNA isolation and cDNA synthesis

Total RNA was extracted from isolated Gastrocnemius muscle tissue using TRIzol reagent (Thermo Fisher Scientific, USA) based on the manufacturer’s protocol. Total RNA concentrations of the samples were evaluated by Nanodrop (Nanolytik, Düsseldorf, Germany) at 260 nm. DNase (Thermo Fisher Scientific) treatment was conducted in order to eliminate probable remaining amount of the genomic DNA in RNA samples. cDNA was synthesized using 1 µg of total RNA immediately after RNA isolation for each sample, by means of PrimeScriptTM RT reagent Kit (TaKaRa, Japan) as stated in respective protocol. cDNA samples were stored at − 80 °C for further use.

### Quantitative real‑time polymerase chain reaction (qRT PCR)

The relative expression levels of target genes were measured by quantitative real-time reverse transcription-polymerase chain reaction (qRT-PCR) and normalized with 18s rRNA as an internal control. The list of the primers is shown in Additional file 1: Table S2. Real-time PCR was carried out by Applied Biosystems StepOnePlus™ instrument (Thermo Fisher Scientific) using SYBR green PCR Master Mix (TaKaRa). Standard cycling protocol was used to perform Real-time PCR. Gene expression assessment was performed using the 2^−ΔΔCT^ method [20].

### Protein extraction and Western blot analysis

Equal amount of Gastrocnemius muscle tissue (~ 10 mg) was homogenized in 1 mL of TRIzol reagent and total protein was extracted according to the manufacturer’s protocol. Running SDS-PAGE 10% was used for PPARGC1α and 15% was used to detect TFAM and GLUT4. The proteins were then transferred to PVDF (Bio-Rad, USA) membrane. The membranes were then blocked by 10% BSA in TBST solution with 0.01% Tween (v/v) for TFAM and GLUT4 and 10% (v/v) BSA + 5% (w/v) skim milk in TBST solution with 0.01% Tween for PPARGC1α at 4 °C overnight. The membranes were incubated with primary antibodies for 1.5 h at room temperature. Primary antibodies were used with the following concentrations: 1:1000 for TFAM (ab131607, Abcam, UK), 1:200 for GLUT4 (ab176245, Abcam, UK), 1:1500 for PPARGC1α (ab191838, Abcam, UK), and 1:2500 for β-Actin (A2228, Sigma, USA). The membranes were washed and incubated with appropriate secondary antibodies, Goat Anti-Rabbit IgG H&L (HRP) (1:20,000, Santa Cruz, USA) and HRP-conjugated goat anti-mouse IgG (1:5000, Dako, Japan P0447), for 1 h at room temperature. Finally, the protein bonds were visualized by an Amersham ECL Advance Western Blotting Detection Kit (GE Healthcare, USA) and the intensity of the bonds were quantitated by image J software (http://rsb.info.nih.gov/ij/).

### Transmission electron microscopy

The samples were fixed by glutaraldehyde 3% (Agar Scientific, UK) in phosphate buffer at pH:7.2 overnight. The samples were then kept in Osmium tetroxide 1% (Agar Scientific, UK) in phosphate buffer pH:7.2 for 1 h. After each step, the samples were washed by 0.1 M phosphate buffer pH:7.2. Next, samples were dehydrated bycethanol and acetone. The samples were then resin infiltrated by spurr’s resin (Agar Scientific), acetone and pure spur. They were polymerized in 70 °C oven and thin sections (80 nm thickness) were prepared using RMC MT-7000 ultramicrotome. The samples were then stained with uranyl acetate and lead citrate and were examined at 50 kV by EM-10, Zeiss, Germany transmission electron microscope.

### Measurement of ATP levels

ATP measurement was carried out by ATP Assay Kit Colorimetric/Fluorometric kit (ab83355, Abcam, USA) according to the manufacturer’s protocol. In summary, nearly 10 mg of Gastrocnemius muscle was washed in cold PBS and homogenized in 100 µL of ice-cold perchloric acid 2 N in order to deproteinizing the tissue. The excessive perchloric acid was precipitated and neutralized by appropriate amount of KOH. The pH was monitored during the neutralization process to reach a 6.5–8 pH range. Standard samples were prepared according to manufacturer protocol. The test samples and background control were prepared in 96 well plate according to manufacturer protocol for colorimetric assay. Finally, the plate was read by a plate reader at OD 570 nm.

### Statistical analysis

All statistical analyses were performed using SPSS software, version 25.0 (SPSS Inc., USA). Real-time PCR and Western blot were repeated three or two times and the final results were expressed as means ± standard error of the mean. Student’s *t*‑test and one-way analysis of variance (ANOVA) with Tukey post-hoc were performed to distinguish statistical significance. Statistical significance was considered as *p* < 0.05. The plots are visualized by Graphpad Prism version 8 (GraphPad Software, San Diego, CA, USA).

## Results

### Herbal component FTIR assay

The transmittance FTIR spectrum of the SGTC herbal component is presented in the Additional file 1: Figure S1. The herbal extract could have antioxidant capacity due to its Phenolic acid and Flavonoid components. The related peaks are presented in the Additional file 1: Figure S1.

### AGE-rich high fat diet leads to obesity and type 2 diabetes

**Fig. 2:**
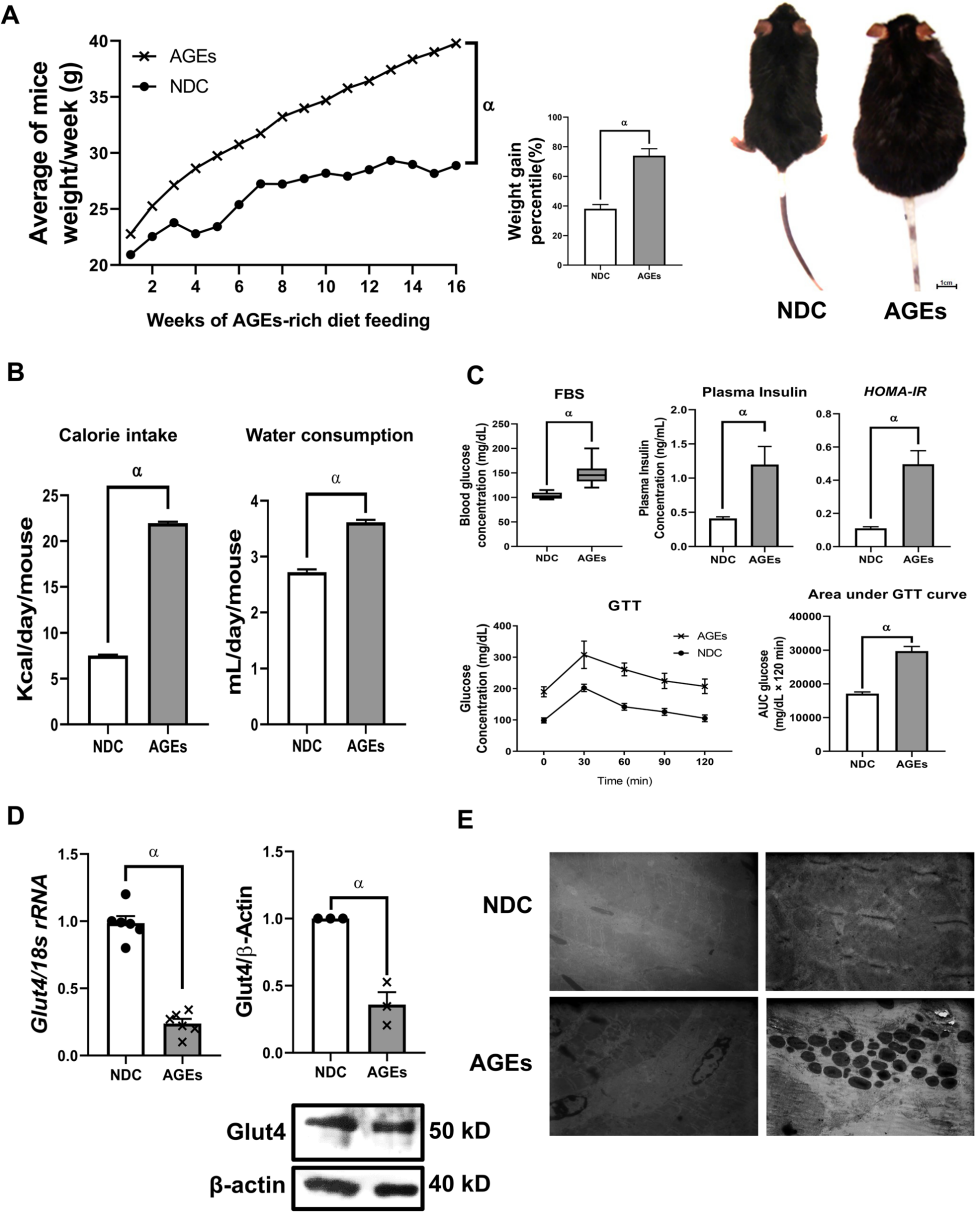
Induction of obesity and T2DM in mice. According to the morphological, biochemical, and molecular results, type 2 diabetes was successfully induced by the AGEs-rich high fat diet in male C57BL/6 mouse model. Average of mice weight and weight gain percentile in AGEs group compared to normal diet controls (NDC) (**A**). Calorie intake and water consumption of NDC and AGEs (**B**). Biochemical results of AGEs-rich high fat diet consumption in comparison with a normal diet, including FBS, plasma insulin, *HOMA-IR* index, GTT, and area under GTT curve (**C**). Glut4 expression in skeletal muscle of NDC and AGEs groups in both transcript and protein level (**D**). Skeletal muscle electron micrograph of mice in NDC and AGEs groups. The muscle ultrastructure in the control group shows normal architecture with regular and ordered A and I bands, Z and M lines. In higher magnification, the mitochondria and their cristae are normal and myofibrillar structure is distinct (upper left × 6300 magnification, upper right × 20,000 magnification). In the AGEs group, rupture of myofibrils and formation of apoptotic bodies were the prominent findings (lower left × 6300 magnification, lower right × 20,000 magnification) (**E**). All values are presented as mean ± SEM. α: statistical significant with NDC group (*p* value < 0.05)

We observed a significant increase in the weight of the AGE-rich high-fat diet fed mice in comparison with NDC (Normal diet control) group which were fed with a standard diet by almost 72% (Fig. 2A). Also, the weight gain percentile after 16 weeks of diet feeding for the AGE group was nearly 37% higher than NDC group (Fig. 2A). Additionally, the amount of consumed food and water of the AGEs mice was significantly higher than NDS groups (Fig. 2B).

The biochemical tests declared the T2DM induction of AGE group. The FBS level of AGE-fed mice was significantly higher than NDC group and it was above the range of normal FBS level i.e. 110 mg/dL. The serum insulin level of AGE was approximately three times more than NDC group, which was statistically significant. The *HOMA-IR* index was significantly higher in AGE compered to NDC indicating insulin resistance in the AGEs group. Finally, the GTT level of AGE was higher than NDC as the area under GTT curve declares (Fig. 2C).

To confirm T2DM in the molecular level, relative expression of *Glut4* in both AGE and NDC groups was measured in both mRNA and protein level. This clarified that the expression of *Glut4* decreased both in mRNA and protein in AGE group by 0.5 fold (Fig. 2D).

The ultrastructure of the muscular cells in the control group appeared normal with regular and ordered A bands, I bands, Z lines and M lines. The normal mitochondria with well-defined cristae arrangement were present in the sarcoplasm adjacent to Z lines (Fig. 2E). Whereas, mitochondrial structure of AGEs group was disordered, i.e. rupture of myofibrils and formation of apoptotic bodies (Fig. 2E).

### SGTC consumption ameliorated the diabetic conditions

**Fig. 3:**
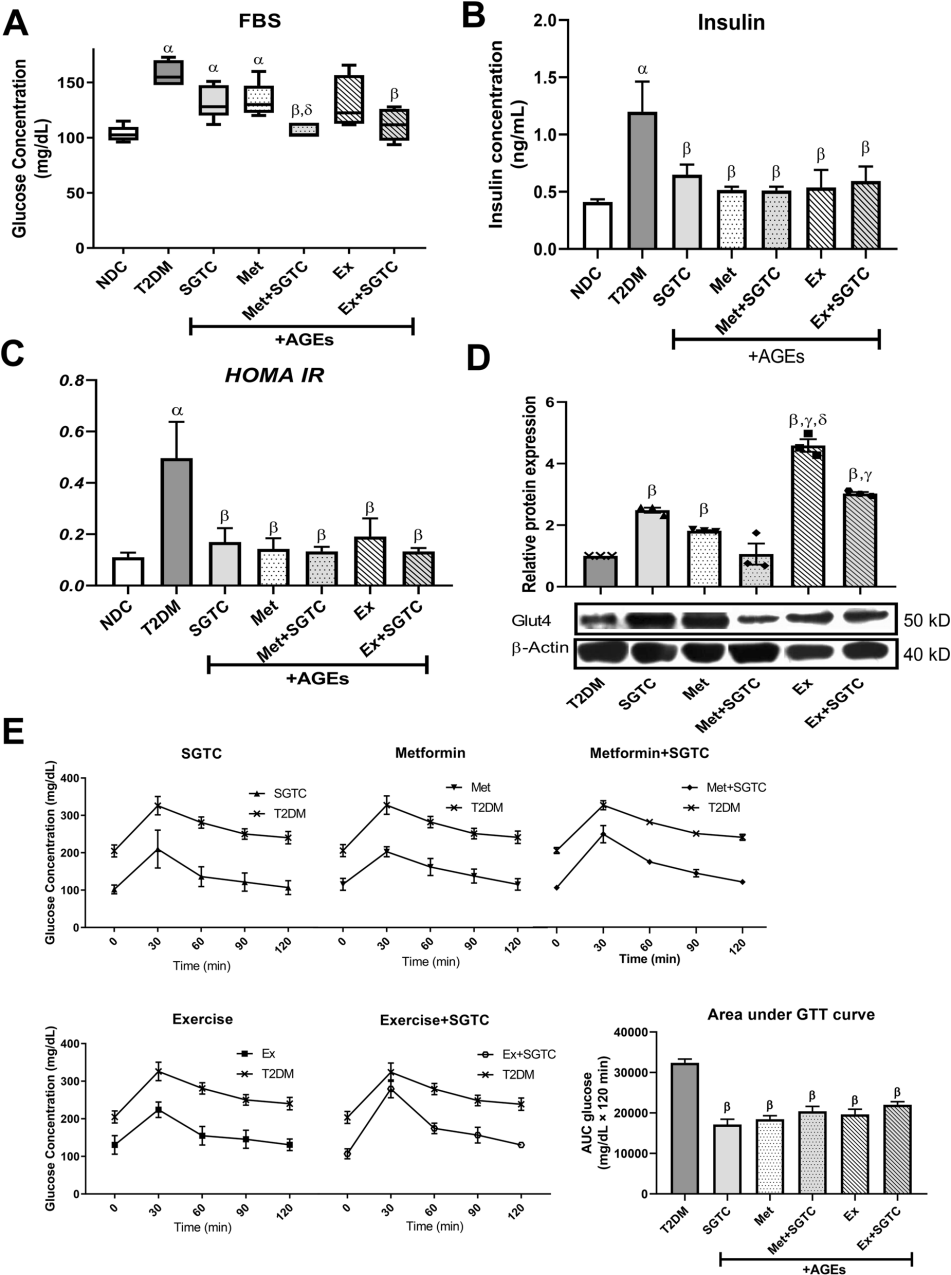
Biochemical and molecular results of different treatments for type 2 diabetes. The biochemical results including FBS (**A**), plasma insulin (**B**), and *HOMA-IR* index (**C**) of different treatments compared to NDC and T2DM groups. Glut4 protein expression of different treatments compared to T2DM in skeletal muscle (**D**). GTT and area under GTT curve of different treatments in comparison with T2DM (**E**). All values are presented as mean ± SEM. α: statistical significant with NDC group (*p* value < 0.05). β: statistical significant with AGEs group (*p* value < 0.05). γ: statistical significant with SGTC group (*p* value < 0.05). δ: statistical significant with Met group (*p* value < 0.05)

The FBS level of both SGTC treated- and exercise treated-mice decreased compared to T2DM mice but the difference was not statistically significant. Although, a statistical significant decrease was observed in the Met + SGTC and Ex + SGTC by approximately 34 and 31% respectively in comparison with T2DM (Fig. 3A). Notably, the insulin level of serum in different treated groups had a same trend and they were all decreased by almost 50% compared to T2DM (Fig. 3B). Next, the *HOMA-IR* index decreased in all of the treatment significantly compared to the diabetic untreated group. No obvious difference was observed between different treatments (Fig. 3C).

To test one of the most important intercellular molecular factors in T2DM, the relative expression level of GLUT4 protein was measured. This protein was upregulated significantly by nearly 2.5 and twofold in the SGTC and Met groups respectively. The Met-SGTC group showed no significant alternation for this protein. Additionally, the protein level of GLUT4 was upregulated by fourfold in Ex and by threefold in Ex-SGTC than T2DM. The protein level of GLUT4 was significantly higher in both Ex and Ex-SGTC compared to both SGTC and Met groups (Fig. 3D).

The GTT results were lower in the treated groups in comparison with T2DM mice. Although the trend of the GTT was not the same in different treatments, but the area under curve (AUC) of GTT of the treated groups showed the same level of decrease compared to T2DM, almost 50% for all of the treated groups compared to T2DM. However, there was no statistical difference among the treated groups in the AUC of GTT either (Fig. 3E).

### Mitochondrial markers were upregulated unlike ATP

**Fig. 4:**
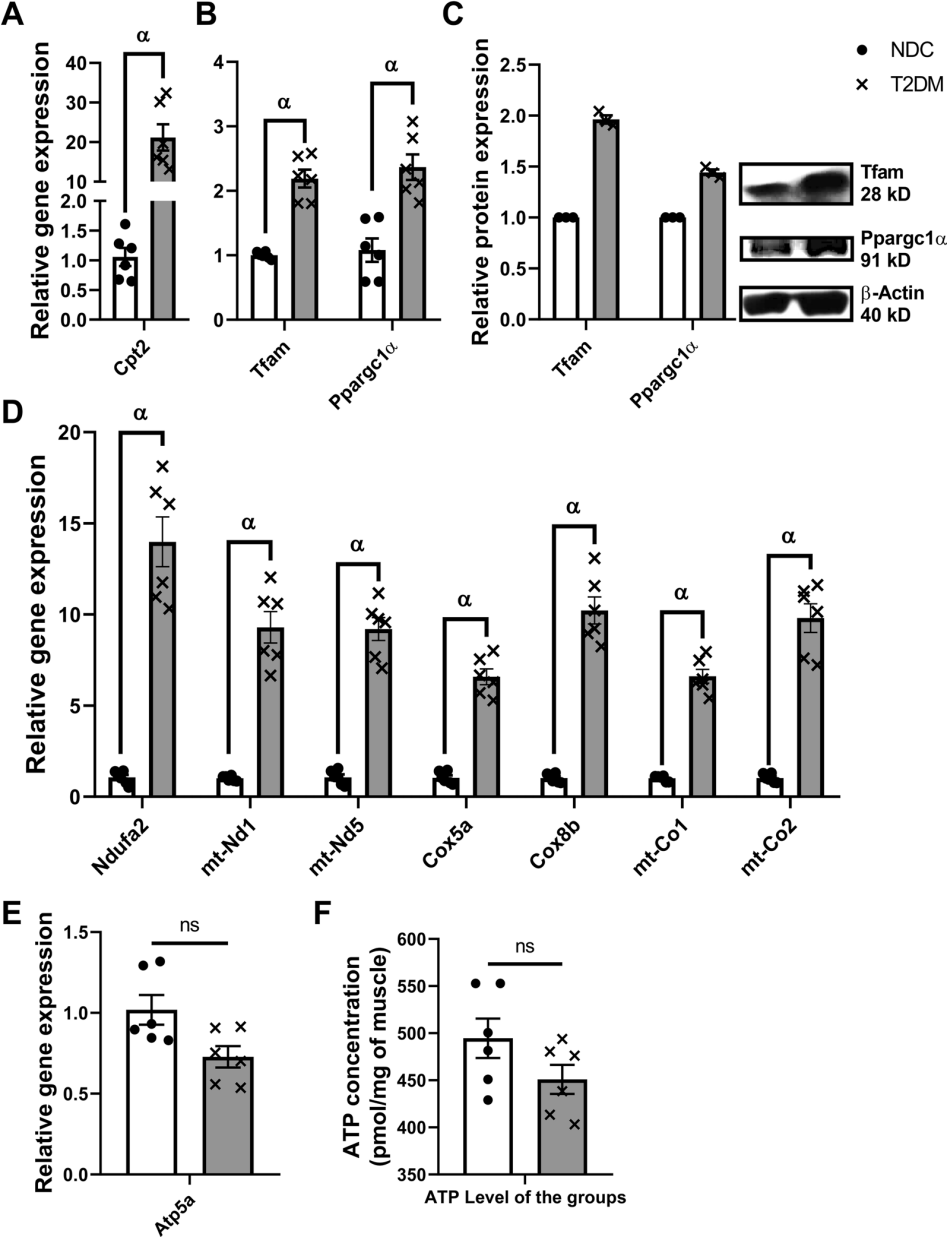
Mitochondrial markers in T2DM versus healthy condition. An upregulation was observed in most of the mitochondrial master regulators and subunit components in the skeletal muscle of diabetic mice (T2DM) compared to normal controls (NDC). The relative expression of *Cpt2*, a β-oxidation marker, in NDC and T2DM (**A**). Mitochondrial biogenesis master regulators in the mRNA (**B**) and protein level (**C**) in T2DM compared to NDC. The relative expression of some of the ETC subunits (**D**). No significant change was observed in *Atp5a* relative expression (**E**) and ATP level of skeletal muscle cells (**F**) among T2DM and NDC groups. All values are presented as mean ± SEM. α: statistical significant with NDC group (*p* value < 0.05). *ns* Not significant (*p* value $\ge$ 0.05)

Transcript levels of several molecular factors of the mitochondria were measured to find the behavior of the mitochondria in response to AGE rich-high fat diet induced type 2 diabetes. Relative mRNA expression of *Cpt2*, one of the main regulators of mitochondrial β-oxidation, was greatly upregulated in T2DM by nearly 20 fold compared to NDC group (Fig. 4A). The major upstream regulators of the mitochondria, *Tfam* and *Ppargc1α* RNA, were also studied in both mRNA and protein level. Both of these markers were upregulated significantly in T2DM compared to NDC (Fig. 4B, C).

Additionally, the mRNA relative expression of some of the main subunits from the first and final complex of electron transport chain, were also measured by real time qPCR. The relative mRNA expression of all of these markers, i.e. *Ndufa2*, *mt-Nd1*, *mt-Nd5*, *Cox5a*, *Cox8b*, *mt-Co1*, and *mt-Co2*, increased significantly increased by at least sevenfold in the T2DM group compared to NDC (Fig. 4D). Unlikely, the relative expression of *Atp5a* and the ATP level of the muscle cells remained almost unchanged in T2DM compared to NDC (Fig. 4E, F).

### Mitochondrial main regulators were modified after SGTC treatment

**Fig. 5:**
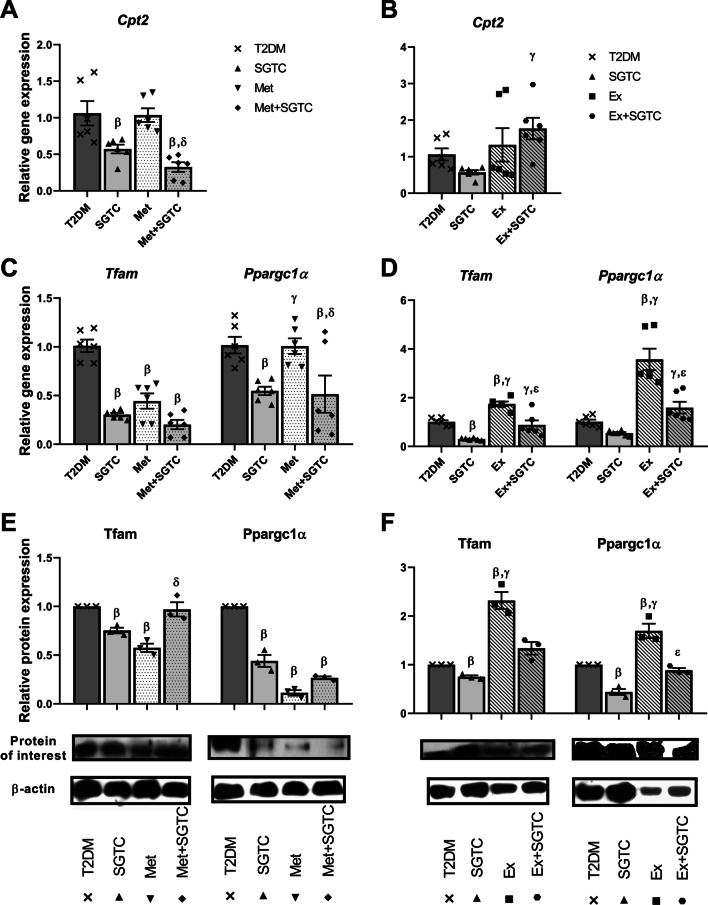
Relative expression of mitochondrial β-oxidation and biogenesis markers after treatments. β-oxidation marker, i.e. *Cpt2*, relative expression after herbal and chemical drugs (**A**) and endurance exercise (**B**) treatments. Transcript level of mitochondrial biogenesis master regulators, *Tfam* and *Ppargc1α,* after herbal and chemical drugs (**C**) and endurance exercise (**D**). Tfam and Ppargc1α relative protein expression after herbal and chemical drugs (**E**) and endurance exercise (**F**) treatments. All values are presented as mean ± SEM. β: statistical significant with AGEs group (*p* value < 0.05). γ: statistical significant with SGTC group (*p* value < 0.05). δ: statistical significant with Met group (*p* value < 0.05). ε: statistical significant with Ex group (*p* value < 0.05)

After treatments with SGTC and exercise trainings, the relative expression of the markers were measured. The relative expression of *Cpt2* decreased significantly due to SGTC and Met + SGTC treatments compared to T2DM, but no significant change was observed in Met group compared to T2DM. Additionally, the mRNA level of *Cpt2* was significantly lower in Met + SGTC compared to Met group (Fig. 5A). In addition, the relative expression of *Cpt2* was significantly higher in Ex + SGTC compared to SGTC, but no other significant differential expression was observed among the treatments (Fig. 5B).

The relative expression of master regulators of mitochondrial biogenesis, both in mRNA and protein level, showed almost similar trend in the treated groups. *Tfam* relative expression was downregulated significantly in SGTC, Met, and Met + SGTC groups compared to T2DM. The relative expression of *Ppargc1α* decreased in SGTC and Met + SGTC groups after treatment, but no changes was observed in the transcript level of the Met group. The relative expression of *Ppargc1α* in the Met group was significantly higher than SGTC and Met + SGTC (Fig. 5C). Moreover, the relative expression of both *Tfam* and *Ppargc1α* increased significantly in Ex group compared to both T2DM and SGTC. On the other hand, no significant change was observed in Ex + SGTC compared to T2DM. Of note, the relative expression of *Tfam* and *Ppargc1α* in the Ex + SGTC group were higher than SGTC and lower than Ex group, this observation was statistically significant (Fig. 5D). Additionally, the protein relative expression of Tfam was downregulated in SGTC and Met groups compared to T2DM, but no significant change was observed in the Met + SGTC group. The relative expression of Tfam in the Met + SGTC group was also significantly higher than Met group. The relative expression of *Ppargc1α* was downregulated significantly in SGTC, Met, and Met + SGTC groups compared to T2DM. Same as mRNA level, the protein level of TFAM and PPARGC1α were significantly upregulated in Ex group compared to T2DM and SGTC. No change was observed in Ex + SGTC compared to T2DM and SGTC in both case of *Tfam* and *Ppargc1α*. The relative expression of *Ppargc1α* was lower significantly in Ex + SGTC versus Ex (Fig. 5F).

**Fig. 6:**
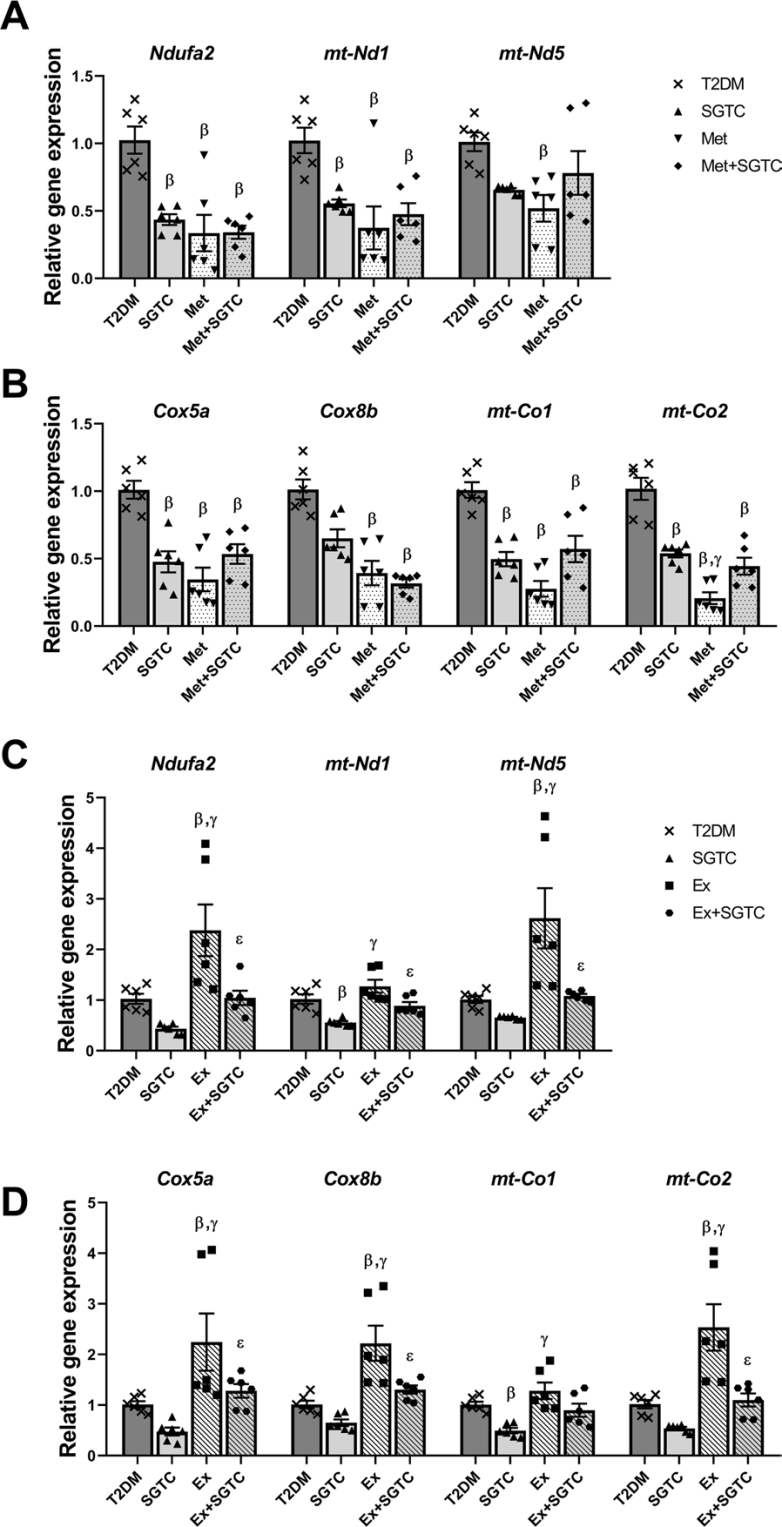
Transcript levels of mitochondrial ETC subunits were modified after treatments. RNA levels of ETC subunits from both nuclear and mitochondrial origins were modified in skeletal muscle after drugs treatments and exercise. Relative transcript expression of ETC complex 1 (**A**) and complex 4 (**B**) subunits after herbal and chemical drug treatments compared to diabetic conditions. ETC complex 1 (**C**) and complex 4 (**D**) subunits relative mRNA expression after endurance exercise treatments. All values are presented as mean ± SEM. β: statistical significant with AGEs group (*p* value < 0.05). γ: statistical significant with SGTC group (*p* value < 0.05). δ: statistical significant with Met group (*p* value < 0.05)

The relative expression of mitochondrial ETC markers were also downregulated significantly as a result of drug treatments, i.e. SGTC, Met, and Met + SGTC. The relative expression of *Ndufa2*, *mt-Nd1*, *Cox5a*, *mt-Co1* and *mt-Co2* were downregulated in SGTC group compared to T2DM. All of the ETC markers, which were investigated in this study, were decreased in Met group in comparison to T2DM. In addition, almost all of these markers, except *mt-Nd5*, decreased in Met + SGTC compared to T2DM (Fig. 6A, B). Moreover, the relative expression of these markers upregulated significantly in the Ex group compared to T2DM and SGTC except for *mt-Nd1* and *mt-Co1*, which this significant upregulation was only observed in comparison with SGTC and NOT for T2DM. Not surprisingly, a significant downregulation was observed in the Ex + SGTC compared to Ex group in all of the markers (Fig. 6C, D).

**Fig. 7:**
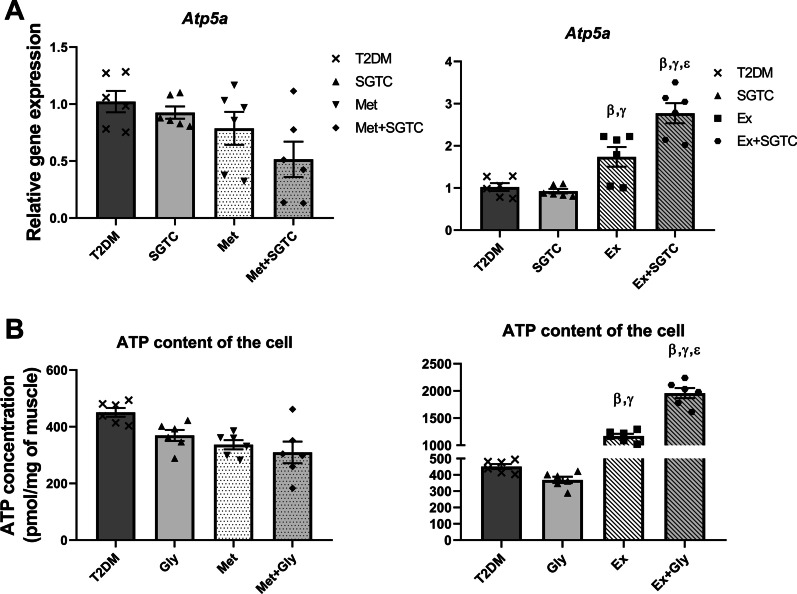
*Atp5a* relative expression and intracellular ATP levels of the cell increased after treatments. *Atp5a* mRNA relative expression (**A**) and ATP content of skeletal muscle cells (**B**) after drug and endurance exercise treatments in comparison with diabetic condition. Significant upregulation was observed both in *Atp5a* relative expression and ATP level of the cells in Ex and Ex + SGTC treatments compared to T2DM. All values are presented as mean ± SEM. β: statistical significant with AGEs group (*p* value < 0.05), γ: statistical significant with SGTC group (*p* value < 0.05), ε: statistical significant with Ex group (*p* value < 0.05)

Finally, the relative expression of *Atp5a* and the ATP level of muscle cells appeared to have similar trend. No significant change was observed in the *Atp5a* mRNA level nor in the ATP concentration of the cells among the drug treated groups, i.e. SGTC, Met, and Met + SGTC compared to the T2DM group. However, a highly significant increase was observed in the relative transcriptome expression of *Atp5a* and ATP concentration of Ex and Ex + SGTC groups compared to T2DM and SGTC. Surprisingly, the highest significant expression level of *Atp5a* and ATP concentration was observed in the Ex + SGTC group. This highly upregulation was statistically significant compared to T2DM, SGTC, and Ex groups (Fig. 7A, B).

## Discussion and conclusion

In the present study, we demonstrated that AGE-rich high fat diet increased mitochondrial marker with no significant change in mitochondrial ATP production. Since the main function of mitochondria is cellular ATP production via oxidative phosphorylation (OXPHOS), the level of ATP can be an index of mitochondrial function [21, 22]. For many years, it has been hypothesized that high fat diet induced IR through mitochondrial dysfunction in the skeletal muscle by decreasing β-oxidation rate, which consequently increases the accumulation of lipid mediators -i.e. CER and DAG- and leads to disruption of insulin signaling [8, 23–25]. In this concept, many studies demonstrated that high fat diet feeding leads to decreasing of β-oxidation enzyme activities, downregulation of Ppargc1α protein expression as the main regulator of mitochondrial biogenesis, and decreasing of oxidative metabolism enzyme activities in diabetic patients [24, 26–28]. In contrast, recent evidence declared that high fat diet not only does not reduce mitochondrial markers level, but actually increase them as well [9, 10]. Our study also revealed that AGE-rich high fat diet increase β-oxidation capacity and most of mitochondrial marker.

The first phase of this study demonstrated that high fat AGE-rich diet resulted in obesity and induction of T2DM. After 2 weeks of adaptation, mice were fed with an AGE-rich high fat diet for 16 weeks to induce obesity and sedentary, major contributor of T2DM. Then different treatments were started for 8 weeks (Fig. 1). As shown in Fig. 2, AGEs group were significantly more obese than NDC. Additionally, the hallmarks of diabetes were observed as a result of AGEs-rich high fat diet. These includes the higher level of FBS, higher level of serum insulin, the increase of *HOMA-IR* index, the increase of GTT, and downregulation of Glut4 in both mRNA and protein level in the AGEs group compared to the NDC, as previously observed in T2DM [29].

In the next phase of this study, we observed a highly significant increase of 20 fold in a β-oxidation marker, i.e. *Cpt2*, almost 100% upregulation in both mRNA and protein level of *Ppargc1α* and *Tfam*, and at least sevenfold increase in most of the oxidative metabolism mRNA level in T2DM group compared to NDC. Since PPARGC1α is the main regulator of mitochondrial biogenesis, it regulates the expression of nuclear encoded mitochondrial proteins, including ETC genes, and regulates the expression of *Tfam*, the main transcription factor for mitochondrial genome [14]. However, despite the upregulation of mitochondrial markers in T2DM, we did not observe any significant change in the rate of ATP production in the diabetic group (Fig. 4). This is because the energy required by diabetic cells is less than β-oxidation rate [22]. We speculated that most of the electron energy in ETC would be consumed for the sake of ROS production, as we observed in our previous study [16]. However, this need to be carefully elucidated in further studies.

In fact, when mitochondria face an enormous FFA upload resulting from high fat diet, it needs to manage that somehow; hence, the cells need to increase the fat oxidation level, which is initiated by increasing CPTs level. Our result showed an upregulation of *Cpt2* mRNA level, which convert long chain acyl carnitine into LCACOA in the inner membrane of mitochondria, in the diabetic group compared to NDC. Turner et al. [9] was the first who confirmed that high fat diet results in an increase of the β-oxidation capacity. Similarly, Hancock et al*.* reported that FFA caused by high-fat diet, are endogenous ligands of PPARɣ and activation of this element following by FFA stimulation would induce the transcription of *Cpt1* promotor, which finally leads to an increase in the capacity of β-oxidation. They also showed that FFA overload and PPARɣ overexpression both result in an increase in PPARGC1α protein expression by a posttranscriptional mechanism [10].

The cells also attempt to overcome FFA overload by increasing the number of mitochondria. Therefore, an increase in mitochondrial biogenesis and oxidative metabolism, that we observed, were suggested by this. However, these increases did not end up with ATP production (Fig. 4) as there was no change in energy demand in the cells. Indeed, in type 2 diabetes induced by a high-fat diet, the β-oxidation rate prevails over the TCA cycle and mitochondrial respiration capacity. This has also been suggested that this process increase mitochondrial ROS formation, which is initiated by electron leakage in the ETC toward oxygen, resulting in the formation of superoxide [22]. That is what we considered as mitochondrial dysfunction. Besides that, we observed no changes in the relative expression of *Atp5a* gene in the T2DM group compared to NDC (Fig. 4E). In fact, a highly similar trend was observed between the expression of *Atp5a* and the ATP concentration of muscle cells. This condition might be another limiting factor for the insufficient ATP production rate in the diabetic group. We hypothesized that the promoter of ATPase complex might be affected by another factor of diabetic condition. This hypothesis is needed to be thoroughly checked in the following studies.

Apart from that, since AGE-rich high fat diet was used in this study, interaction of these compounds with their receptors (RAGE) leads to activation AGEs-RAGE axis, which triggers the activation of oxidative stress and inflammatory pathways by activation of NADPH oxidase and NFκB [5]. This mechanism finally leads to mitochondrial dysfunction [6, 30]. Evidence have shown that these processes occur more rapidly in diabetic patients compared to healthy individuals [4]. Therefore, AGE compound trigger oxidative stress pathway and leads to ROS production. This excessive ROS production, which comes from the AGEs-RAGE axis, may intensify the mitochondrial dysfunction, which results from high fat overload, in long term.

ROS cause a series damages in the cells. These damages force the cells to initiate some preventive actions, which includes mitophagy or, under high stress levels, even apoptosis [31], as observed to be initiated in some of the cells of the diabetic group in the electron microscopy images (Fig. 2). Removing a part of mitochondria by mitophagy could reduce the mitochondrial copy number of the cells. In the long term, that leads to substrate oxidation decrease. Besides, in long-term, β-oxidation decrease can lead to ATP rate decrease [32]. Moreover, insulin deficiency, after progression of TDM, is often associated with a lack of ATP production [33]. Therefore, the present theories about substrate-oxidation reduction in diabetic patients could also make sense. Probably in the long term, as beta-oxidation decreases, the cells would have further aggravating lipid accumulation and disrupting of AKT-GLUT4 pathways [8]. In this study, the mice were fed with an AGEs-rich high fat diet only for 24 weeks. By elongation of this period, a decrease in substrate-oxidation levels and in the mitochondrial copy number might be probable.

As mentioned earlier, we observed a significant upregulation of mitochondrial markers, as a result of high fat diet with no sufficient functionality; this condition might be a result of ROS formation due to AGE-rich high fat diet in long term. According to Bonnard et al. [30] mitochondrial dysfunction might not be the initial step in skeletal muscle oxidative capacity reduction and in fact ROS production was the trigger of mitochondrial dysfunction. In conclusion, we suggest that initially the cells tries to overcome the FFA overload by increasing the number of mitochondria, but due to excessive ROS damage, as a result of FFA accumulation and AGEs-RAGE axis activation, the cells tries to eliminate the insufficient mitochondria in long term as presented in most of the theories [23, 24].

In the next step, we investigated the effects of different treatments to enhance mitochondrial markers and ATP production rate. Investigating the effect of the herbal components on mitochondrial markers showed that SGTC decreased the overexpression of mitochondrial markers (Figs. 5A, C, E, 6A, B), on the other hand, the ATP level remained unchanged in the SGTC group compared to T2DM (Fig. 7A). The same trend was observed in the Met and Met-SGTC groups. This may be due to thermogenic fat burning activities by Ucp3 in muscle, as there was no change in the energy demand and ATP needs of the cells. Some studies have shown an increase in Ucp3 expression after ingestion of Salvia and Ginseng extract (two components of SGTC) and Metformin [34–36]. Additionally, SGTC and Met antioxidant capacity might also improve the functional role of mitochondria by ROS reduction according to our previous study (Additional file 1: Figure S2) [13, 16, 37, 38].

On the other hand, it seems that exercise upregulated all mitochondrial markers significantly in comparison with T2DM (Figs. 5D, F, 6C, D). It is well recognized that endurance exercise training have major impacts on mitochondrial content and function in skeletal muscle [14]. Approximately, 4–7% of muscle cells volume is consisted of mitochondria and the amount of that vary over a considerable range by endurance exercise [39]. Endurance exercise is involved in the biochemical makeup of muscle cells and the skeletal muscle, as a highly adaptive organ, responds gradually to this variable by increasing *Ppargc1α* levels [14]. Overexpression of *Ppargc1α* in skeletal muscle results in increasing mitochondrial content and GLUT4 expression (Fig. 3D) [14]. Therefore, as expected, the expression of most mitochondrial markers increased in the Ex group because of *Ppargc1α* upregulation. Moreover, PPARGC1α promotes GLUT4 expression directly by activating the myocyte enhancer factors (MEF), MEF2C and MEF2A and indirectly through NRF1 [40]. This action finally leads to recruitment of GLUT4 containing vesicles from cytoplasm toward the cell surface and it mediates the transporter of glucose [14]. Eight weeks of endurance exercise in this study, could lead to dramatic increase in the expression of mitochondrial markers and improve the rate of ATP production.

On the contrary, in Ex + SGTC group, the expression of mitochondrial markers and the levels of Ppargc1α and Tfam proteins were almost reduced compared to the Ex group except Cpt*2* (Figs. 5B, D, F, 6C, D). This downward trend in both gene and protein expression appears to be due to SGTC; however, the molecular function of each herbal component of SGTC has not yet been fully determined and further research is crucial. Nevertheless, many studies have demonstrated the antioxidant properties of each component of the drug [13, 38]. Endurance exercise enhances the mitochondrial function and ATP production through Ppargc1α and Tfam pathways whereas SGTC may act by ameliorating the expression of mitochondrial markers and its antioxidant properties [16]. It is likely that the herbal component in Ex + SGTC group, decreased the rate of ROS and significantly improved the rate of ATP production and mitochondrial overall functionality compared to the Ex group (Fig. 7B). We also observed an overall improvement in T2DM complications such as FBS, plasma insulin, *HOMA-IR*, and GTT, in Ex + SGTC group (Fig. 3). This may also show a probable enhancement of glucose uptake, which resulted in more effective ATP production when the energy demand of the cell is high due to exercise.

In the present study, we induced type 2 diabetes by AGEs-rich high fat diet in male C57BL/6 mice model. We observed a significant upregulation of mitochondrial markers in response to diabetic conditions, which is thought to have occurred to overcome fat overload. As this increase did not follow by energy demand, it seems that it finally led to ROS production. To test any improvement in diabetic complications, an herbal component (SGTC) and endurance exercise training were investigated. Both SGTS and exercise treatments ameliorated the complications to some extent. Although a greater improvement was observed in exercise along with SGTC treatment. Overall, it seems that Ex + SGTC can be proposed as a possible option for further clinical studies. However, some limitations should be mentioned. As we only used male C57BL/6 mice in a small sample size, repetition of these studies in female mice with a larger sample size could be more reliable, to study sex-specific parameters for T2DM and treatment modalities. As mitochondria plays an important role in metabolic disorders, its copy number and other mitochondrial markers, including Ucp3 expression could be interesting parameters for further studies. Additionally, further investigations are needed to study the exact molecular mechanism of the drug components and its combination with exercise in muscle and other T2DM related organs.

## Conclusion

Overall, we concluded that AGEs rich high-fat diet makes an increase in β-oxidation capacity and mitochondrial marker. Regular exercise along with SGTC consumption might be very helpful in improving mitochondrial function and thereby in ameliorating diabetes complications.

## Supplementary Information

**Additional file 1. Supplementary Table 1.** Endurance exercise protocol. Exercise sessions were applied on a motorized treadmill with a 0° incline. (~ 70% vO_2_ max). **Supplementary Table 2.** Primers list for the genes of interests. **Supplementary figure 1.** FTIR spectra of herbal component (SGTC). Several antioxidant hallmarks were observed in the FTIR spectra of SGTC herbal component, including “Aromatic compounds”, “Anhydride”, and “Phenol”.

## Data Availability

All of the raw data and the rest of the materials are remained in Royan Institute for Biotechnology and are available upon request.

## Abbreviations

AGEs: Advanced glycation end products
ANOVA: Analysis of variance
AUC: Area under curve
CER: Ceramide
DAG: Diacylglycerol
FBS: Fasting blood sugar
FFAs: Free fatty acids
FTIR: Fourier-transform infrared spectroscopy
GTT: Glucose tolerance test
HED: Human equivalent dose
HFD: High fat diet
HOMA-IR: Homeostatic Model Assessment for Insulin Resistance
I.P.: Intra-peritoneal
IR: Insulin resistance
NDC: Normal diet control
qRT PCR: Quantitative real‑time polymerase chain reaction
RAGE: Receptor of AGEs
SGTC: Salvia, Ginseng, Trigonella, and Cinnamon
SM: Skeletal muscle
T2DM: Type 2 diabetes mellitus
