# Correction: The combination of endurance exercise and SGTC (Salvia–Ginseng–Trigonella–Cinnamon) ameliorate mitochondrial markers’ overexpression with sufficient ATP production in the skeletal muscle of mice fed AGEs-rich high-fat diet

**DOI:** 10.1186/s12986-022-00676-2

**Published:** 2022-07-08

**Authors:** Maryam Haghparast Azad, Iman Niktab, Shaghayegh Dastjerdi, Navid Abedpoor, Golbarg Rahimi, Zahra Safaeinejad, Maryam Peymani, Farzad Seyed Forootan, Majid Asadi-Shekaari, Mohammad Hossein Nasr Esfahani, Kamran Ghaedi

**Affiliations:** 1 ACECR Institute of Higher Education, Isfahan, Iran; 2 Department of Animal Biotechnology, Cell Science Research Center, Royan Institute for Biotechnology, ACECR, Isfahan, Iran; 3 Department of Cell and Molecular Biology and Microbiology, Faculty of Biological Science and Technology, University of Isfahan, Hezar Jerib Ave., Azadi Sq., P.O. Code 81746-73441 Isfahan, Iran; 4 Department of Biology, Faculty of Basic Sciences, Shahrekord Branch, Islamic Azad University, Shahrekord, Iran; 5 Legal Medicine Research Center, Legal Medicine Organization, Tehran, Iran; 6 Neuroscience Research Center, Neuropharmacology Institute, Kerman University of Medical Sciences, Kerman, Iran

## Correction: Nutrition & Metabolism (2022) 19:17 https://doi.org/10.1186/s12986-022-00652-w

Following publication of the original article [1], the authors would like to correct the heading and the name of the electron microscope.

The incorrect heading is: Transmittance electron microscopy

The correct heading is: Transmission electron microscopy

The incorrect name of the electron microscope is: Zeiss EM900

The correct name of the electron microscope is: EM-10, Zeiss, Germany

The original article [1] has been corrected.
