# Supplementary material for: The combination of endurance exercise and SGTC (Salvia–Ginseng–Trigonella–Cinnamon) ameliorate mitochondrial markers’ overexpression with sufficient ATP production in the skeletal muscle of mice fed AGEs-rich high-fat diet
Source: Nutr Metab (Lond). 2022 Mar 5;19:17. doi: 10.1186/s12986-022-00652-w (PMC8897771; doi:10.1186/s12986-022-00652-w)
Supplement: Supplementary file 1 — Additional file 1. Supplementary Table 1. Endurance exercise protocol. Exercise sessions were applied on a motorized treadmill with a 0° incline. (~ 70% vO2 max). Supplementary Table 2. Primers list for the genes of interests. Supplementary figure 1. FTIR spectra of herbal component (SGTC). Several antioxidant hallmarks were observed in the FTIR spectra of SGTC herbal component, including “Aromatic compounds”, “Anhydride”, and “Phenol”. [file 12986_2022_652_MOESM1_ESM.pdf]

Supplementary table 1

Week	Speed (m/min)	Exercise duration/session (min)	Exercise sessions/week
Adaptation	7-10	10	5
Firs	7, 10, 13, 10, 7	10, 3, 19, 3, 10	5
Second	7, 10, 13, 15, 13, 10, 7	5, 3, 3, 23, 3, 3, 5	5
Third	10, 13, 16, 17, 16, 13, 10	5, 3, 3, 23, 3, 3, 5	5
Fourth	10, 13, 16, 18, 16, 13, 10	5, 3, 3, 23, 3, 3, 5	5
Fifth	10, 13, 16, 19, 16, 13, 10	5, 3, 3, 23, 3, 3, 5	5
Sixth	10, 13, 16, 20, 16, 13, 10	5, 3, 3, 23, 3, 3, 5	5
Seventh	10, 13, 16, 18, 21, 16, 13, 10	5, 3, 3, 3, 20, 3, 3, 5	5
Eighth	10, 13, 16, 19, 22, 16, 13, 10	5, 3, 3, 3, 20, 3, 3, 5	5

Supplementary table 2

mRNA	Product length	Primer's sequence
18s-rRNA	85 bp	F: 5′- CGGACACGGACAGGATTG -3′<br>R: 5′-TCGCTCCACCAACTAAGAAC -3′
Ppargc1α	161 bp	F: 5’-CCCTGCCATTGTTAAGACC-3′<br>R: 5’-TGCTGCTGTTCCTGTTTTC-3′
Tfam	92 bp	F: 5’-CTTCAACCACCACACCACT-3′<br>R: 5’-AATCTCTAAGCCTCCTCAATACAA-3′
Cpt2	142 bp	F: 5’-CATTGACGCCATTCAGTTTCAG-3′<br>R: 5’-GCAGGATTCATAGGTAGCCAC-3’
Ndufa2	151 bp	F: 5`- TGTGAGGGATTTCATCGTGC -3`<br>R: 5`-CAGATTGTTCAGAGACACCGT -3`
Cox5a	166 bp	F: 5`-ACATATTTCAACAAGCCAGAC-3`<br>R: 5`-TAACAACCTCCAAGATGCG-3`
Cox8b	99 bp	F: 5`-GTTCCCAAAGCCCATGTCTC-3`<br>R: 5`-AACCATGAAGCCAACGACTATG-3`
mt-Nd1	120 bp	F: 5′- GACAGAAGGAGAATCAGAATTAG -3′<br>R: 5′- ATAGTTGTTAGGGCGTTTATTAG -3′
mt-Nd5	165 bp	F: 5′- ATTGCCTTCTCTACATCAAGC -3′<br>R: 5′- GATGTCTTGTTCGTCTGCC -3′
mt-Co1	100 bp	F: 5′- ATTACTATACTACTAACAGACCG -3′<br>R: 5′- CAAAGAATCAGAACAGATGC -3′
mt-Co2	123 bp	F: 5′- CCGTTAAAACCATAGGGCAC -3′<br>R: 5′- CTAGCAGTCGTAGTTCACCAG -3′
Atp5a	112 bp	F: 5′- TCCGCTTACATTCCAACA -3′<br>R: 5′- ACACAGACAAACCCACAT -3′
Glut4	166 bp	F: 5′- CTGAAGGATGAGAAACGGAAGT -3′<br>R: 5′- CGAAGATGCTGGTTGAATAGTAGA -3′

Supplementary figure 1

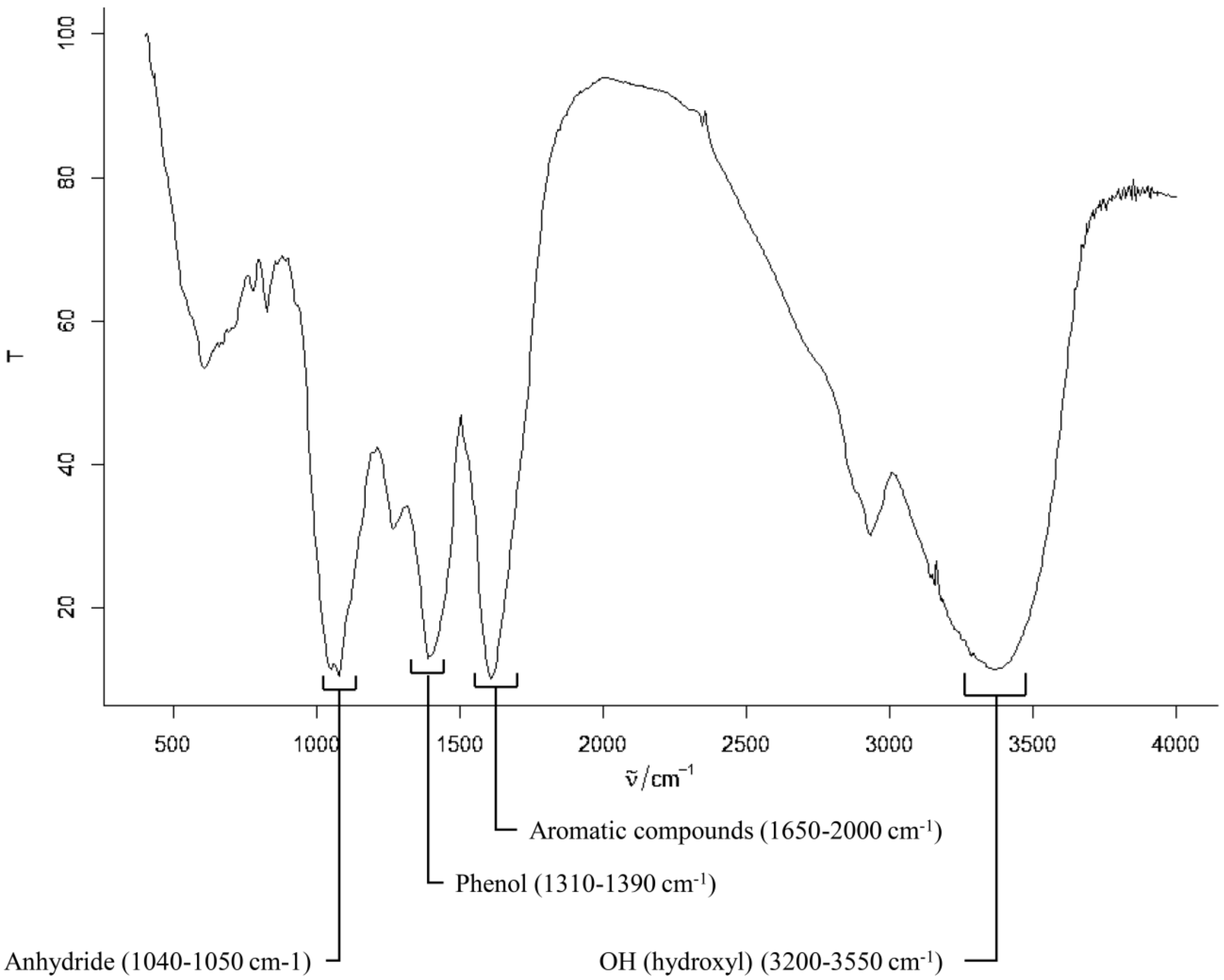

Supplementary Table 1. Endurance exercise protocol. Exercise sessions were applied on a motorized treadmill with a 0° incline. (~ 70% vO_2 max).

Supplementary Table 2. Primers list for the genes of interests.

Supplementary figure 1. FTIR spectra of herbal component (SGTC). Several antioxidant hallmarks were observed in the FTIR spectra of SGTC herbal component, including "Aromatic compounds", "Anhydride", and "Phenol".
